# Morphophysiology of Potato (*Solanum tuberosum*) in Response to Drought Stress: Paving the Way Forward

**DOI:** 10.3389/fpls.2020.597554

**Published:** 2021-01-15

**Authors:** Dominic Hill, David Nelson, John Hammond, Luke Bell

**Affiliations:** ^1^School of Agriculture, Policy and Development, University of Reading, Reading, United Kingdom; ^2^Branston Ltd., Lincoln, United Kingdom

**Keywords:** drought, stress tolerance, climate change, crop morphophysiology, food security, potato, *Solanum tuberosum* L., high-throughput phenotyping

## Abstract

The cultivated potato (*Solanum tuberosum* L.) is currently the third most important food crop in the world and is becoming increasingly important to the local economies of developing countries. Climate change threatens to drastically reduce potato yields in areas of the world where the growing season is predicted to become hotter and drier. Modern potato is well known as an extremely drought susceptible crop, which has primarily been attributed to its shallow root system. This review addresses this decades old consensus, and highlights other, less well understood, morphophysiological features of potato which likely contribute to drought susceptibility. This review explores the effects of drought on these traits and goes on to discuss phenotypes which may be associated with drought tolerance in potato. Small canopies which increase harvest index and decrease evapotranspiration, open stem-type canopies which increase light penetration, and shallow but densely rooted cultivars, which increase water uptake, have all been associated with drought tolerance in the past, but have largely been ignored. While individual studies on a limited number of cultivars may have examined these phenotypes, they are typically overlooked due to the consensus that root depth is the only significant cause of drought susceptibility in potato. We review this work, particularly with respect to potato morphology, in the context of a changing climate, and highlight the gaps in our understanding of drought tolerance in potato that such work implies.

## Introduction

### Potato Cultivation

The cultivated potato, *Solanum tuberosum*, originated in the New World, where its wild relatives can still be found from the southern United States (38°N) to Argentina and Chile (41°S) (Spooner et al., 2004). Potato cultivation began in South America around 8,000 years ago (Lutaladio and Castaldi, 2009), resulting in the many thousands of landraces still grown by Andean smallholders (Bradshaw and Ramsay, 2009). Potatoes were first introduced to Europe in the 16th century by Spanish conquistadors during the Columbian exchange (Lutaladio and Castaldi, 2009). By the end of that century, potatoes had been introduced into the United Kingdom and Ireland, where they had a transformative effect on society, helping to feed the industrial revolution (Bradshaw and Ramsay, 2009). Records of potato breeding in Europe begin around a 100 years later in 1807 (Bradshaw and Ramsay, 2009), but overreliance on a few cultivars and clonal propagation resulted in the infamous destruction of the Irish potato crop by late blight in 1845 (Lutaladio and Castaldi, 2009). A concerted effort to produce resistant, high-yielding cultivars followed, some of which are still grown today (Lutaladio and Castaldi, 2009).

Between 2012 and 2016, potato rose from the fourth (Monneveux et al., 2013) to the third (Aliche et al., 2018) most important food crop in the world, behind only rice and wheat (Camire et al., 2009). As of 2017, potatoes are grown on about 19.3 million hectares globally, with an estimated total yield of 388 million tonnes (FAOSTAT, 2018). Over half of the global potato harvest now comes from developing countries (Mackay, 2009), where potatoes are an important source of employment, income and nutrition (Lutaladio and Castaldi, 2009). The production of potatoes in developing countries increased from 1.5 to 21.1 million tonnes in the half century between 1961 and 2011 (FAOSTAT, 2018). Potato is a favored crop in developed and developing countries alike as it yields more food, more efficiently than any other crop (Lutaladio and Castaldi, 2009). Approximately 85% of the biomass of a potato plants is edible: much higher than the 50% of edible biomass from cereals (Lutaladio and Castaldi, 2009). Consequently, potatoes are the most productive food crop per unit area in the world, yielding 5600 kcal/m^3^: over double that of wheat (2300 kcal/m^3^) (Renault and Wallender, 2000).

### Potato Water Use in a Changing Climate

Potatoes are a relatively water-efficient crop, producing more calories per unit water used than any other crop (Sun et al., 2015). A kilogram of potatoes requires only 105 l kg^–1^ of water to produce, compared to 1408 l kg^–1^ for rice, 1159 l kg^–1^ for wheat and 710 l kg^–1^ for maize (Renault and Wallender, 2000). However, in the United Kingdom and the United States, potatoes are often supplemented with additional water, particularly in the drier areas of eastern England (Daccache et al., 2011), and the warmer southern states (Byrd et al., 2014), respectively. In some regions of the Mediterranean, including southern Italy, irrigation for early crops is essential to obtain marketable yields (Cantore et al., 2014). In a typical dry year, maincrop potatoes in the United Kingdom need between 143 and 313 mm of irrigated water, depending primarily on the agroclimatic zone and secondarily on the local soil available water content (Knox et al., 1997). In the exceptionally dry year of 2018, the minimum estimated irrigation requirement for maincrop potatoes in the United Kingdom increased to 154 mm (Knox and Hess, 2019). These irrigation requirements are higher than most other crops grown in the United Kingdom including sugar beet, 0 to 253 mm; cereals, 0 to 82 mm; carrots, 0 to 258 mm; and strawberries, 0 to 132 mm (Knox et al., 1997). Only apple orchards are estimated to require a greater volume of irrigated water in a typical dry year than potatoes, needing 114 to 364 mm, depending on agroclimatic zone and soil type (Knox et al., 1997). In the southern state of Florida in the United States, potato production typically uses 10 mm of water every 24–36 h between flowering and harvest, totaling around 610 mm (Byrd et al., 2014).

Despite their water efficiency, this high water requirement makes potatoes are extremely susceptible to drought stress throughout their life cycle (Schafleitner et al., 2009). The susceptibility of potato to drought has primarily been attributed to its shallow root system (van Loon, 1981), with cultivar root length being correlated with yield under drought condition (Lahlou and Ledent, 2005); and canopy characteristics (Aliche et al., 2018), with stem-type canopy cultivars performing better under drought conditions than leaf-types (Schittenhelm et al., 2006). These characteristics can result in dramatically decreased yields under drought conditions, with one study reporting a 87% decrease in tuber number in the cultivar Désirée, which was unable to maintain stem height and leaf number under drought stress, both characteristics of stem-type cultivars (Luitel et al., 2015).

As potatoes are such a drought susceptible crop (Schafleitner et al., 2009), climate change represents a real threat to potato production in the United Kingdom and around the world. Regional climate changes are being brought about by global warming and its effects on weather systems at planetary, regional and local levels (Arnelf and Reynard, 1996). The specific effects of a global increase in average temperature on local weather patterns are unpredictable but, the incidence of extreme and adverse weather conditions are likely to increase, with significant effects on crop production (Harkness et al., 2020). In the United Kingdom, precipitation is likely to be redistributed throughout the year, with droughts in the summer and extreme rainfall in the winter both becoming more frequent (Rial-Lovera et al., 2017). Climate change has been predicted to slightly increase potato production in the United Kingdom as higher temperatures speed plant development and lengthen the growing season (Daccache et al., 2011). However, due to water unavailability, the land area suited to unirrigated potato production in the United Kingdom is predicted to decrease by 74–95%, depending on future emissions (Daccache et al., 2012). Historically rainfed areas will have to be irrigated in the future, increasing water demand and production costs much more than small increases in water use by already irrigated areas (Daccache et al., 2012). Current irrigation infrastructure will be insufficient to meet peak water supply needs in ∼50% of years (Daccache et al., 2011), leading to reduced yields, increased costs and possible crop failures (Daccache et al., 2012).

### Potato Research

Despite the global popularity of potato, and its importance as a source of employment, income, and nutrition, there is a distinct lack of recent morphophysiological potato research. In the case of drought, the majority of studies investigating its effects on root growth (Tourneux et al., 2003; Lahlou and Ledent, 2005; Mane et al., 2008), canopy growth (Jefferies and MacKerron, 1987; Jefferies, 1993a; Deblonde and Ledent, 2001; Lahlou et al., 2003), and yield (Levy, 1986b; Jefferies and MacKerron, 1989) are at least 10 years old. There have been recent studies, published in the last five years, observing the effects of drought on the morphophysiology of potato (Aliche et al., 2018; Chang et al., 2018; Michel et al., 2019; Pourasadollahi et al., 2019), but such studies are limited in the scientific literature. Unlike in other crops, including tomato (Susič et al., 2018), grape (Zovko et al., 2019), and maize (Asaari et al., 2019), there has been even less research investigating the effects of drought on potato using modern phenotyping methods, such as multispectral, hyperspectral or three-dimensional imaging. In a review published in 2013 regarding drought tolerance in potato, the mean year of publication for citations that demonstrated the measurement of drought-related phenotypic responses in potato was 2001 (Monneveux et al., 2013).

The reasons for the recent disinterest in morphophysiological research in potato are unclear but may result from a feeling within the field that the effects of a specific abiotic stress on the morphophysiology of potato have been completely elucidated, or a shift in focus to the molecular and genetic components underlying these traits and responses, which have previously been reviewed (Obidiegwu et al., 2015). In the case of potato and drought, the majority of morphophysiological studies were published between the late 1980s and early 2000s (MacKerron and Jefferies, 1986; Jefferies and MacKerron, 1987, 1989, 1993; Deblonde and Ledent, 2001; Lahlou et al., 2003; Tourneux et al., 2003; Lahlou and Ledent, 2005), establishing a consensus regarding the effects of drought stress on potato. While these studies form the foundation of the field, they were obviously limited by contemporary technology. These studies primarily focused on traits that could be easily measured at the time, including tuber number (MacKerron and Jefferies, 1986; Deblonde and Ledent, 2001), plant height (Deblonde and Ledent, 2001; Tourneux et al., 2003), and dry matter metrics (Jefferies and MacKerron, 1987, 1993). Thus, a revivification of the field that takes advantage of modern cultivars, novel agricultural practices, and high-throughput phenotyping (HTP) techniques is called for, making use of innovative methodologies, including the functional phenomics pipeline (York, 2019), to investigate potato morphophysiology with unprecedented precision.

Functional phenomics, the study of plant phenotypes as they relate to plant function under specific environmental conditions, aims to address the significant knowledge gap between the ever-advancing field of plant genetics and plant morphophysiology, which remains a limiting factor in our understanding of plant performance in an agronomic setting (York, 2019). Recent advances in imaging technologies at a range of wavelengths make this process orders of magnitude more practical as HTP platforms allow the generation of vast quantities of spectral data with much lower temporal and manual input (Kim et al., 2020). Previous research investigating a specific phene, an individual genetically determined phenotypic trait (York et al., 2013) for example, canopy openness, relied on manual measurements of variables including stem height, individual leaf area, and stem and leaf dry weights (Schittenhelm et al., 2006). Now, a properly calibrated multispectral sensor could capture this data in seconds, alongside measures of chlorophyll conductance, water status, and vegetation indices (Kim et al., 2020).

By accelerating the rate at which desirable phenes can be identified, investigated, and understood, HTP platforms have the potential to relieve the current bottleneck in plant breeding cycles (Araus and Cairns, 2014). This is essential as a doubling of global crop production is predicted to be necessary by 2050 (Tilman et al., 2011), an increase which current crop yield improvement rates will be unable to meet (Ray et al., 2013). HTP platforms will be an important tool in the process of accelerating crop improvement rates, although there is a risk that the generation of such vast datasets will shift the breeding cycle bottleneck from phenotyping to data analysis (Cobb et al., 2013). However, advances in machine learning and data mining will likely alleviate this problem, elucidating relationships between agronomically relevant variables and compound indices which are currently too abstract to investigate (Araus and Cairns, 2014). Presently, simple regression analysis of the data captured by HTP platforms can also be used to discover discrete phenes that associate with agronomic traits under specific environmental conditions (York, 2019). However, due to the stigmatization of data mining for hypothesis-generation, and obvious conceptual reasons, a broader understanding of crop morphophysiology as it relates to a specific environmental stress is necessary. Thus, this review is an attempt to synthesize the field as it stands, paving the way forward for morphophysiological potato research that takes advantage of developments in functional phenomics.

## Methodology

An initial literature search was conducted with Web of Science, using the search terms “*S. tuberosum*” and “drought.” “*S. tuberosum*” was used instead of “potato,” or any variation thereof, to exclude references to sweet potato, *Ipomoea balatas*. The results of this search (*n* = 520) were then filtered using the Web of Science agronomy category to exclude the many biochemical, genetic, and physiological studies that have been well-covered elsewhere (Obidiegwu et al., 2015). The remaining references (*n* = 110) were further filtered using Web of Science categories to exclude proceedings papers and book chapters, leaving only primary research articles (*n* = 105). The further exclusion of studies where the effects of drought on potato morphophysiology were, for our purposes, confounded by the experimental manipulation of other variables, including plant nutrition and ambient temperature, was based on the title and abstract of each paper (*n* = 23). This search found few but mostly recent studies. Thus, the remaining references included were found either as references in the papers returned by the Web of Science search, or by using the “Cited by…” hyperlink for the older papers on Google Scholar (*n* = 70). These references were subject to the same inclusion and exclusion criteria as the initial search.

As stated previously, there has been little recent research regarding the morphophysiology of potato under drought-stressed conditions, particularly concerning the investigation phenotypes that are hypothesized to confer any level of drought-tolerance. The results of the literature search ranged from 1958 to 2020 with a mean publication year of ∼2001. A large number of the references reviewed (*n* = 18) were published in the ten years between 1986 and 1995, inclusive, when interest in in potato morphology was being extensively studied by the household names of potato research including R. A. Jefferies, D. Levy, C. D. van Loon, D. K. MacKerron, and P. C. Struik. However, twenty-five references were found that were published between 2011 and 2020, inclusive, fourteen of which were published in the last five years. These more recent studies are often investigating various genetic and biochemical markers of drought, but warrant inclusion here as they also include relevant measurements, for our purposes, including tuber fresh weight, number and dry matter, which remain inescapable due their commercial significance.

## Effects of Drought on Potato

### Effects of Drought on Potato Growth

Drought is technically a purely meteorological term that describes a prolonged period of time with little or no rain (Solh and Van Ginkel, 2014). From a biological perspective, the definition of drought is expanded to include its effects on plant life. Drought in this context is still a period of little or no rain, but one which leads to a soil moisture deficit and, consequently, a reduction of water potential in affected plant tissues (Mitra, 2001). In agriculture, drought may be considered as a period of water shortage that leads to a moisture deficit in the soil and drought stress in a crop, preventing the crop from reaching its maximum genetic potential yield (Mitra, 2001). Drought stress is a crop’s response to drought and includes the morphological and physiological adaptations that occur when plants perceive the loss of enough water to maintain pre-drought growth (Jaleel et al., 2009). The effects of drought on potato, discussed below, are technically the result of a plant-initiated response to an environmental change which causes the plant to prioritize survival and reproduction over optimum growth and yield.

Drought may further be defined in terms of onset and duration with respect to a crop’s life cycle. Intermittent drought describes one or more periods of an inadequate water supply for optimum growth that occur at any time throughout the growing season (Neumann, 2008). After intermittent drought, soil moisture is restored allowing normal growth to resume. This differs from terminal drought, which also describes a period of inadequate water supply for optimal growth, but one from which there is no replenishment of soil moisture within the crop’s life cycle (Neumann, 2008). Terminal drought causes a progressive decline in soil moisture and, depending on its severity and duration, may result in reduced yields and even early plant death (Neumann, 2008).

Drought stress occurs when plants lose, or perceive the loss of, enough water to maintain optimal growth (Jaleel et al., 2009). Plants generally respond to moderate drought stress with the closure of stomata to reduce further water loss via evapotranspiration (Keskin et al., 2010). This response also reduces gas exchange through the stomata, limiting CO_2_ availability for photosynthetic assimilation (Cornic, 2000; Pourasadollahi et al., 2019). Stomatal closure was previously believed to be a primarily hydraulic response to a decrease in leaf water potential caused by an excessive loss of water by evapotranspiration, regardless of root water potential or soil moisture (Kramer, 1988). However, in many plants, including potato, stomatal closure occurs before any drop in leaf water potential is detectable (Jefferies and MacKerron, 1989; Davies and Zhang, 1991). While hydraulic mechanisms likely do have some role in regulating stomatal conductance (Davies and Zhang, 1991), chemical processes have been shown to regulate stomatal conductance even before any detectable change in leaf water potential (Davies and Zhang, 1991).

Abscisic acid (ABA) has been identified as a key molecule involved in root-to-shoot signaling of a drought stress and as an important regulator of stomatal conductance in wheat (Ali et al., 1998), maize (Bahrun et al., 2002) and soybean (Liu et al., 2003). Potato roots tips have been shown to produce ABA as a response to a moderate decrease in soil moisture (Liu et al., 2005). A linear relationship between xylem-borne ABA, the concentration of which is increased by ABA production in the roots, and stomatal conductance has been observed at mild soil water deficits in potato (Liu et al., 2005). This suggests that chemical root-to-shoot signaling has an important role in stomatal conductance even before detectable decreases in leaf water potential. But, the relationship between ABA and stomatal conductance is less significant at severe soil water deficits (Liu et al., 2005), implying the presence of other unknown mechanisms involved in regulating stomatal conductance in potato. The relationship between ABA and drought tolerance in potato has recently been confused further by evidence that suggests that, only one of two drought-tolerant cultivars, Gwiazda is hypersensitive to ABA signaling, closing its stomata significantly earlier when treated directly with ABA, compared to both, the drought-tolerant cultivar, Tajfun and, the drought-susceptible cultivar, Oberon (Boguszewska-Mańkowska et al., 2018). This suggests the presence of multiple mechanisms contributing to the drought tolerance or susceptibility of potato cultivars (Boguszewska-Mańkowska et al., 2018), some of which remain unknown.

It is also possible that, at higher moisture deficits the above effects reduce a potato plant’s ability to mount an appropriate adaptive response to prevent further water loss. More severe drought stress, or desiccation, has increasingly significant effects on plant cell structure and function as water loss increases (Jaleel et al., 2009). Intense drought stress can cause damage to cellular structure by reducing turgor pressure (Moore et al., 2008), decreasing enzymatic activity involved in adenosine triphosphate (ATP) production and carbon fixation (Farooq et al., 2009), and ultimately plant death (Munné-Bosch and Alegre, 2004). While drought stress is an undesirable response in agronomic terms, it is important to note that drought stress facilitates adaptive mechanisms which evolved as prophylaxes against the above effects at the expense of maximum yields (Basu et al., 2016).

The effects of drought on potato growth vary greatly depending on the cultivar-specific canopy and root characteristics described below. The effects of drought stress on potato also depend on abiotic factors including the duration, timing (Jefferies and MacKerron, 1993) and severity (Stark et al., 2013) of water stress, the implementation of which has never been standardized, as shown in Tables 1–3. Existing soil moisture (Jefferies and MacKerron, 1993), nutrient availability (Saravia et al., 2016) and evaporative demand (Jefferies and MacKerron, 1993) further complicate the effects of drought on potato growth. However, drought represents one of the most essential biological challenges to all crop species (Shao et al., 2009). Thus the effects of drought on fundamental potato plant growth are relatively consistent with small differences between cultivars of primarily agronomic significance (Schittenhelm et al., 2006).

**TABLE 1:** A summary of the effects of drought stress on key physiological root traits in potato and the range of methodologies by which these variables were manipulated.

**References**	**Albiski et al., 2012**	**Anithakumari et al., 2011**	**Haverkort et al., 1990**	**Lahlou and Ledent, 2005**	**Mane et al., 2008**	**Tourneux et al., 2003**
Observations	Decreased root length.	Increased root dry mass.	Decreased stolon number.	Increased root depth, increased root dry mass (Remarka, Nicola and Monalisa), decreased root dry mass (Désirée), increased stolon number.	Decrease root dry mass (Ccompis), no effect on root dry mass (Sulla).	Increased root dry mass.
Cultivar	SY-C.1, SY-C.2, SY-C.3, SY-C.14, SY-C.28, SY-C.29, SY-C.31, SY-C.46, SY-C.52, SY-C.53, SY-C.54, SY-C.55, SY-C.56, SY-C.57, SY-C.58, SY-C.59, SY-C.60, SY-C.61	A random subset of the C × E diploid potato mapping population.	Radosa, Bintje.	Remarka, Dérirée (field and greenhouse); Nicola, Monalisa (field only).	Sullu (subsp. *andigenum*), Ccompis (subsp. *andigenum*).	Alpha, Waycha (subsp. *andigenum*), Luky (subsp. *andigenum*), Ajahuiri (*Solanum ajanhuiri*), Janko Choquepito (*Solanum curtilobum*), CIP 382171.10 (subsp. *tuberosum* × subsp. *andigenum*).
Culture method	*In vitro*	*In vitro*	Field and Pots.	Field (Remarka, Désirée, Nicola and Monalisa) Pots (Remarka and Désirée).	Field	Pots
Drought conditions	Six variations of growth media containing 0, 2, 4, 6, 8, or 10% (w:v) sorbitol to create graduated water potentials between -0.58 MPa (least sever water stress) to −2.5 MPa (most severe water stress).	Water potential of growth media lowered to −0.7 MPa by the addition of polyethylene glycol (PEG) for 7 weeks. 3 of 7 replicates were then allowed to recover for 4 weeks.	Irrigated to field capacity when soil moisture exceeded 100 kPa.	Rainfed in the field. Irrigated to field capacity when soil moisture dropped below −0.8 MPa in the pots.	Irrigated as controls until 45 days after planting when irrigation was completely suspended for 59 days (unclear if drought was terminal or intermittent).	Plants irrigated as controls until being subjected to either intermittent drought (gradual decline in water supply for 5 weeks, and 1 week with no water supply followed by full restoration of water supply) or terminal drought (same as intermittent drought but with no restoration of water supply) at tuberization.
Control		Plants were grown in the same growth media in the absence of PEG. Water potential unclear.	Irrigated to maintain soil moisture levels at “near field capacity” constantly.	Irrigated with 20 mm five times throughout the season in the field. Irrigated to field capacity when soil moisture dropped below −0.3 MPa in the pots.	Irrigated to maintain soil moisture between 0 and −0.02 MPa.	Irrigated to field capacity twice per week.

#### Effects of Drought on Below Ground Growth in Potato

The effects of drought on below ground potato growth are well studied, but these studies often find seemingly contradictory results (Table 1). Drought has been shown to increase maximum root depth (Steckel and Gray, 1979; Lahlou and Ledent, 2005) which, logically, allows potato plants access to deeper soil water (Stalham and Allen, 2004). Total root length, on the other hand, has been found to decrease (Albiski et al., 2012), remain consistent, and increase (Boguszewska-Mańkowska et al., 2020) in response to water stress. Similarly, root dry mass has been observed to increase (Tourneux et al., 2003; Lahlou and Ledent, 2005; Anithakumari et al., 2011), decrease (Lahlou and Ledent, 2005; Mane et al., 2008) and remain constant (Mane et al., 2008) under drought conditions. Stolon number has also been found to both increase (Lahlou and Ledent, 2005) and decrease (Haverkort et al., 1990) due to drought stress.

These apparent contradictions are likely due to differences between cultivar genotype x environment (GxE) responses to drought (Rudack et al., 2017; Boguszewska-Mańkowska et al., 2020), which become exaggerated with increasing water stress (Epstein and Grant, 1973). Experimental variation, including differences in drought severity, duration and timing; location, soil type, and tuber physiological age, contribute to these conflicting results (Steckel and Gray, 1979; Obidiegwu et al., 2015). Root growth in potato is also particularly susceptible to soil compaction which reduces root depth and density (Stalham et al., 2007), preventing potato from more extensively foraging for water and nutrients (White et al., 2005). Due to the unpredictable effects of these factors on root growth during drought and challenges in quantifying root growth accurately, it may be more productive to focus on above ground growth to reduce water stress.

#### Effects of Drought on Above Ground Growth in Potato

Canopy growth is one of the most drought sensitive biological processes in plants (Shao et al., 2009) and is a result of the irreversible elongation of many individual plant cells (Lockhart, 1965). This process is reliant on the maintenance of high turgor pressure, which stretches plant cell walls causing cell expansion and thus plant growth (Szabolcs, 1999). Consequently, when the fraction of transpirable soil water falls below a species specific threshold, leaf growth ceases (Schafleitner, 2009). In most crops, leaf growth stops when the transpirable soil water drops below 40–50% (Weisz et al., 1994), but in potato, leaf growth declines with 60% of transpirable water remaining in the soil, highlighting its sensitivity to drought stress (Weisz et al., 1994). Thus, the first noticeable effect of drought stress on potato is reduced leaf growth (Jefferies and MacKerron, 1987), resulting in potato canopy growth being more affected by drought stress than root growth (Boguszewska-Mańkowska et al., 2020). Drought also typically decreases both the individual leaf area (Jefferies, 1993b; Kesiime et al., 2016) and number of green leaves (Deblonde and Ledent, 2001) in potato, as well as reducing potato stem number (Lahlou et al., 2003; Chang et al., 2018) and height (Deblonde and Ledent, 2001; Chang et al., 2018), although the latter is less affected in early cultivars (Deblonde and Ledent, 2001). Through these mechanisms, the evidence for which is summarized in Table 2, drought reduces the photosynthetic area of the canopy: the primary determinant of productivity in potato (Allen and Scott, 1980).

**TABLE 2:** A summary of the effects of drought stress on key physiological canopy traits in potato and the range of methodologies by which these variables were manipulated.

**References**	**Aliche et al., 2018**	**Chang et al., 2018**	**Deblonde and Ledent, 2001**	**Jefferies, 1993b**	**Jefferies and MacKerron, 1987**	**Lahlou et al., 2003**
Observations	Decreased canopy growth rate, fewer new leaves, premature leaf shedding.	Decreased stem length.	Reduced leaf number, reduced stem height.	Reduced individual leaf area.	Reduced leaf growth.	Reduced stem number.
Cultivar	103 commercial cultivars.	Chubaek, Superior, Jayoung.	Eersteling, Jaerla, Krostar Eersteling, Claustar, Bintje, Nicola, Désirée.	19 commercial cultivars.	Maris Piper, Record, Désirée, Pentland Crown, Pentland Dell, Pentland Squire.	Remarka, Dérirée (field and greenhouse); Nicola, Monalisa (field only).
Culture method	Field	Field	Field	Field	Field	Field (Remarka, Désirée, Nicola and Monalisa) Pots (Remarka and Désirée).
Drought conditions	Rainfed.	Rainfed until emergence and then totally deprived of water until tuberization. After tuberization, plants were irrigated when indicators of drought stress were visible (“wilting and growth retardation”).	Deprived of water by a plastic sheet at 50% emergence for 8 weeks.	Deprived of water by a mobile rain shelter from emergence to harvest.	Deprived of water by polythene sheeting laid over the plants from emergence to harvest.	Rainfed in the field. Irrigated to field capacity when soil moisture dropped below −0.8 MPa in the pots.
Control	Rainfed plus irrigated with roughly 15 to 30 mm of water on 14 occasions.	Predominantly rainfed, irrigated with trickle irrigation between May and June during a dry period. Plants were irrigated when indicators of drought stress were visible (“wilting and growth retardation”).	Rainfed only or rainfed plus 37 and 35 mm of irrigation in 1996 and 1996, respectively.	Rainfed plus sprinkler irrigation to maintain a soil moisture deficit of <25 mm.	Rainfed plus trickle irrigation to maintain a soil moisture deficit of <30 mm.	Irrigated with 20 mm five times throughout the season in the field. Irrigated to field capacity when soil moisture dropped below −0.3 MPa in the pots.

In a recent and comprehensive study investigating the effects of drought on 103 cultivars of potato, the response of canopy growth to drought stress was found to be highly variable (Aliche et al., 2018). Generally, naturally occurring periods of drought reduced canopy growth regardless of drought timing (Aliche et al., 2018). This is a logical result of a lack of water inhibiting plant growth: the product of high turgor pressure forcing cell expansion (Szabolcs, 1999). Early drought was found to slow canopy growth, increasing the time taken for plants to reach optimum canopy cover (Aliche et al., 2018). Later drought had a greater effect on maximum canopy cover due to reduced new leaf formation and early shedding of mature leaves (Aliche et al., 2018). Early drought has also recently been demonstrated to slow canopy development by reducing stem length by 75 to 78%, further increasing time to full canopy cover (Chang et al., 2018). This result was replicated over two growing seasons, and in one of the two study years, stem thickness and stem number were also found to be significantly decreased by drought (Chang et al., 2018). The lack of statistical significance in the first trial year was likely due to a shorter drought period which, crucially, ended before emergence when stem number is effectively fixed, baring the death of individual stems (Chang et al., 2018). The significant results from the following year corroborate older findings regarding the negative effects of drought on stem length (Deblonde and Ledent, 2001) and number (Lahlou et al., 2003) in potato.

Another recent study found that drought significantly reduced the leaf area index (LAI) of three cultivars, Karaka, Moonlight, and Russet Burbank, subjected to drought for the duration of the life cycle (Michel et al., 2019). Droughted plants were irrigated with a fifth of the volume of water supplied to well-watered plants to prevent early senescence (Michel et al., 2019). Each cultivar was affected similarly, with drought stress reducing LAI from the end of the first month after planting (Michel et al., 2019). Except for cv. Karaka, LAI started to decline earlier under drought conditions compared to well-watered conditions (Michel et al., 2019), reducing leaf area duration (LAD) and thus the total radiation intercepted throughout the life cycle: the primary determinant of dry biomass production in potato (Allen and Scott, 1980). This finding was recently corroborated in the cultivars Desirée and Karú INIA, where water restriction was found to have a greater negative effect on tuber yield than high temperatures, due to the effects of drought stress on LAD (Ávila-Valdés et al., 2020). LAI was also found to decrease in the cv. Banba under drought conditions, although it is unclear how LAI or LAD were affected in this cultivar over the course of the life cycle (Pourasadollahi et al., 2019). These findings corroborate previous work that suggested that LAD, rather than the maximum LAI at a single point, was most strongly associated with biomass production, most of which is partitioned to tubers (Jefferies and MacKerron, 1993). The differences seen in LAI between cultivars may be due to differences in canopy architecture (Michel et al., 2019), which will be discussed later in this review.

The timing of drought has varying effects on different cultivars, particularly with respect to maturity classes (Aliche et al., 2018; Chang et al., 2018). As late maturing cultivars generally require longer to reach exponential canopy growth and full canopy cover, compared to early maturing cultivars (Aliche et al., 2018), late droughts are effectively earlier in the life cycle of late cultivars. This may be indicative of an ability of late maturing cultivars to recover after late droughts by delaying achievement of full canopy cover, which has previously been suggested (Romero et al., 2017). By taking longer to achieve full canopy cover, the relatively large canopies of late maturing cultivars may be less affected by the canopy reduction effects of late drought, allowing these cultivars to recover post-drought and compensate for lost growth. The relatively large canopies of late maturing cultivars have been demonstrated to persist for much longer than similarly droughted early maturing cultivars, increasing LAD, which likely accounts for the significantly higher yields found in late maturing cultivars under drought stress (Aliche et al., 2018).

The cumulative effects of drought stress on above ground potato growth are a result of a reduction in the rate of photosynthesis within the leaves (Pieters and El Souki, 2005). Drought stress affects photosynthesis by limiting ribulose bisphosphate (RuBP) production (Tezara et al., 1999). RuBP production is affected by reduced ATP synthesis, which is inhibited by the high intracellular ionic concentration caused by the low relative water content of leaves during drought stress (Lawlor, 2002). Reduced photosynthetic carbon assimilation leads to the unavailability of substrates required for respiration and plant growth (Flexas et al., 2006). Reduced CO_2_ concentration in the mesophyll due to stomatal closure may also decrease photosynthetic rate in plants during drought (Cornic, 2000). The relative effects of stomatal closure and reduced RuBP production under drought conditions are debated (Parry et al., 2002). Decreased RuBP production has been suggested as the primary effect of drought on growth in soybean (Vu et al., 1987) and sunflower (Tezara et al., 1999). In several other species, including common vine grape and common bean, stomatal conductance has been suggested as the factor limiting plant growth under drought (Bota et al., 2004). The growth limiting effect of drought stress may be species, or even cultivar, specific and vary depending on relative soil water content (Bota et al., 2004) and remains unstudied in potato.

### Effects of Drought on Potato Yield

As the primary outcome of agronomic significance, the effects of drought on tuber yield have been more extensively studied. Tuber yields after drought stress have been quantified in several ways, including total fresh tuber mass (Lahlou et al., 2003; Carli et al., 2014), total tuber dry matter (Deblonde et al., 1999), marketable tuber yield (Steckel and Gray, 1979; Cantore et al., 2014) and tuber number (Deblonde and Ledent, 2001). In general, all these metrics are reduced by drought (Obidiegwu et al., 2015), with some exceptions (Nadler and Heuer, 1995). This review will focus on tuber fresh mass and tuber dry matter and concentration as these are the most economically relevant measures of yield.

#### Effects of Drought on Fresh Tuber Mass

Fresh tuber yields are primarily dependent on tuber dry matter and water content (Jefferies and MacKerron, 1993). Tuber water content and radiation interception are the morphophysiological traits most affected by drought stress in potato (Jefferies and MacKerron, 1987). Fresh potato tubers have a water content of around 80%, with a small amount of variation between cultivars (Navarre et al., 2009). This makes fresh tuber mass highly vulnerable to drought stress, having been shown on more than one occasion to be a greater contributor to yield loss than tuber number (Struik and Van Voorst, 1986; Carli et al., 2014). The vast majority of previously reviewed evidence showing significantly decreased fresh tuber mass after drought (Obidiegwu et al., 2015).

Total water deprivation from emergence to harvest can reduce relative tuber water content of Maris Piper by 69%, compared to potatoes irrigated with sufficient water to maintain a soil water deficit of no greater than 30 mm (Jefferies and MacKerron, 1989). The effects of drought on fresh tuber mass appear to be highly cultivar-dependent (Lahlou et al., 2003). Fresh tuber yield reductions in a single study ranged from 11 to 44% in Désirée and Remarka, respectively, (Lahlou et al., 2003). In this study, field grown potatoes were not totally deprived of water, receiving 148 mm of effective rainfall across the season, which may account for lower yield losses than those observed in totally water deprived Maris Piper (Jefferies and MacKerron, 1989). Many different protocols have been used to assess the effects of drought stress on potato tubers, as shown in Table 3, making it difficult generalize the effects of drought stress on potato, even within cultivars.

**TABLE 3:** A summary of the effects of drought stress on key physiological tuber traits in potato and the range of methodologies by which these variables were manipulated.

**References**	**MacKerron and Jefferies, 1986**	**Martin et al., 1992**	**Painter and Augustin, 1976**	**Steckel and Gray, 1979**	**Tourneux et al., 2003**	**Jefferies and MacKerron, 1987**	**Jefferies and MacKerron, 1989**	**Jefferies and MacKerron, 1993**	**Lahlou et al., 2003**	**Lefèvre et al., 2012**	**Levy, 1986b**	**Luitel et al., 2015**
Observations	Decrease in tuber number	No effect on processing quality, no effect on prevalence of internal or external defects	Increase in prevalence of misshapes	Reduced tuber dry matter	Decreased dry matter concentration (Up-to-Date and Troubadour), increased dry matter concentration (Alpha)	Decreased total dry matter, increased dry matter concentration.	Reduced tuber water content.	Reduced tuber dry matter concentration.	Reduced fresh tuber yield.	Increased tuber water content.	No effect on prevalence of misshapes, increase in prevalence of misshapes (Kondor).	Decreased tuber number.
Cultivar	Maris Piper	Russet Burbank	Russet Burbank	King Edward, Pentland Crown, Majestic, Maris Piper	Alpha, Waycha (subsp. *andigenum*), Luky (subsp. *andigenum*), Ajahuiri (*Solanum ajanhuiri*), Janko Choquepito (*Solanum curtilobum*), CIP 382171.10 (subsp. *tuberosum* × subsp. *andigenum*)	Maris Piper, Record, Désirée, Pentland Crown, Pentland Dell, Pentland Squire.	Maris Piper.	21 Commercial cultivars.	Remarka, Dérirée, Nicola, Monalisa.	21 Andean cultivars	Blanka, Kondor, Draga, Monalisa, Alpha, Désirée, Romano, unnamed clone, Cara.	Five CIP clones, the German cultivar NPI-106, Désirée.
Culture method	Greenhouse plots	Field	Field	Field	Pots	Field	Field	Field	Field (all), Pots (Remarka and Désirée).	Outdoor controlled plots.	Field	Field
Drought conditions	Twenty-two treatments with varying lengths (8 to 40 days) of total water deprivation at either 50% emergence, tuber initiation or small tuber stage.	Six treatments trickle irrigated with 30 mm when SMD reached 50 mm. Irrigation was removed and rain was excluded at various points during the season with varying severity.	Four water treatments. (1) Soil moisture was depleted to 25% before irrigation during early tuber set and then depleted to 65% before irrigation for the remained of the season. (2) Soil moisture was depleted to 65% before irrigation. (3) Soil moisture was depleted to 75% before irrigation. (4) Soil moisture was depleted to 85% before irrigation. How much water was given is unclear but assumed to restore field capacity.	Deprived of water by a mobile rain shelter from emergence to harvest, except for one bout of irrigation with 25 mm of water at the time of tuber formation.	Plants irrigated as controls until being subjected to either intermittent drought (gradual decline in water supply for 5 weeks, and 1 week with no water supply followed by full restoration of water supply) or terminal drought (same as intermittent drought but with no restoration of water supply) at tuberization.	Deprived of water by polythene sheeting laid over the plants from emergence to harvest.	Deprived of water by a mobile rain shelter from emergence to harvest.	Deprived of water by a mobile rain shelter from emergence to harvest.	Rainfed in the field. Irrigated to field capacity when soil moisture dropped below −0.8 MPa in the pots.	As control but irrigation stopped for 58, 86 days after planting.	Irrigated every 3 to 4 days to replace either 0.64 to 0.89 or 0.40 to 0.67 times water lost to evapotranspiration.	Irrigated once soon after planting, then total water deprivation.
Control		Three treatments trickle irrigated with either 20 mm of water when soil moisture deficit reached 30 mm, 30 mm when SMD reached 50 mm or 50 mm when SMD reached 50 mm.		Rainfed plus irrigation to field capacity when the soil moisture deficit reached 25 mm.	Irrigated to field capacity twice per week.	Rainfed plus trickle irrigation to maintain a soil moisture deficit of <30 mm.	Rainfed plus trickle irrigation to maintain a soil moisture deficit of <30 mm.	Rainfed plus sprinkler irrigation to maintain a soil moisture deficit of <25 mm.	Irrigated with 20 mm five times throughout the season in the field. Irrigated to field capacity when soil moisture dropped below −0.3 MPa in the pots.	Deprived from rainfall by a plastic rain shelter and 60 cm below-ground barrier. Drip irrigated to maintain a soil water potential between 0 and −0.02 MPa.	Irrigated every 3 to 4 days to replace water lost by evapotranspiration.	Rainfed and furrow irrigated when soil moisture dropped below 8% to maintain “ideal moisture conditions (8–16%)”.

In contrast, some Andean potato cultivars have been demonstrated to significantly increase tuber water content during drought stress (Lefèvre et al., 2012). This may be due an adaptive drought response that increases tissue K^+^ concentrations, which improve osmotic regulation of tuber water content (Khosravifar et al., 2008). K^+^ supplementation has been shown to promote sucrose storage despite lower assimilate production due to drought stress which may further contribute to osmotic regulation and tuber fresh weight in drought tolerant landraces (Allison et al., 2001). These Andean cultivars are the exception and represent a subspecies of cultivated potato, S. *tuberosum* subsp. *andigenum*, genetically distinct from commercially cultivated cultivars (Raker and Spooner, 2002). However, the Andean population is an important source of genetic variation for use in commercial S. *tuberosum* subsp. *tuberosum* breeding programs (Sukhotu and Hosaka, 2006). As maintaining tuber water content is a key trait associated with yield maintenance under drought conditions (Jefferies and MacKerron, 1989), these cultivars may prove useful in the future.

#### Effects of Drought on Total Tuber Dry Matter

Tuber dry matter correlates with, and is used as a proxy for, yield and quality in potato (Dull et al., 1989). As total dry matter production in potato is proportional to total intercepted radiation (Allen and Scott, 1980), drought stress indirectly reduces tuber dry matter production by reducing the photosynthetic area of the canopy (Jefferies and MacKerron, 1989). Dry matter concentration is clearly highly dependent on tuber water content (Jefferies and MacKerron, 1987) and is most commonly used as an index of tuber quality, especially for processing cultivars (Pritchard and Scanlon, 1997). Low dry matter concentrations in processing cultivars are also associated with higher production costs (Pritchard and Scanlon, 1997). Total tuber dry matter is a more important marker for total yield as it indicates the efficiency of assimilate translocation into tubers (Jovanovic et al., 2010). As such economically significant markers, total dry matter and dry matter content have been extensively investigated in potato. An early study found consistent decreases in tuber dry matter, after total post-emergence water deprivation, in cultivars Pentland Dell, Majestic, Maris Piper, and King Edward (Steckel and Gray, 1979). These represent a range of reputed drought sensitivities, including the drought tolerant Pentland Crown, and drought susceptible King Edward (Steckel and Gray, 1979). The decreases in total dry matter due to drought stress were remarkably similar between these two cultivars: 15.2 to 7.0, and 15.5 to 6.7 t ha^–1^, respectively, (Steckel and Gray, 1979). However, the reported drought tolerance of Pentland Crown was found to be due to its ability to maintain dry matter in marketable tubers, defined as >40 mm in length (Steckel and Gray, 1979). This showed a need to investigate many variables in many cultivars to fully understand the effects of drought stress on total dry matter, especially in the context of marketable output.

Nineteen cultivars of potato, totally deprived of water from emergence to harvest, had a 52% higher tuber dry matter concentration, on average, than plants irrigated to maintain a maximum soil moisture deficit of 25 mm (Jefferies and MacKerron, 1993). The drought stressed plants also had, averaged across all cultivars, 44% less tuber dry matter than the irrigated plants (Jefferies and MacKerron, 1993). This supports the suggestion that increased tuber dry matter concentration is likely a function of reduced water content after drought, rather than of higher dry matter production (Jefferies and MacKerron, 1989). However, while every cultivar showed an increased dry matter concentration after drought, not all cultivars had reduced total dry matter; in cultivars Baillie, Duke of York and Ulster Scepter changes in total dry matter were statistically insignificant (Jefferies and MacKerron, 1993). The authors propose this results from already low total dry matter in these cultivars under irrigation but, Draga, the second lowest yielding cultivar with irrigation, did show significant reductions in total dry matter due to drought stress (Jefferies and MacKerron, 1993). This hypothesis was disputed by later evidence that demonstrated that some cultivars have the potential to produce relatively high dry matter yields under drought stress, despite performing relatively poorly under well-watered conditions (Steyn et al., 1998). Any inherent differences in drought tolerance of these cultivars, which could account for the insignificant changes, were not acknowledged. Baillie, Duke of York and Ulster Scepter are classified, respectively, as “medium-to-high,” “medium,” and “high” drought resistant cultivars by The European Cultivated Potato Database (2008, 2011, 2018) and so should be expected to maintain total tuber dry matter yields under drought stress.

These results contrasted with previous research which found that, while dry matter concentration significantly decreased in cultivars Up-to-Date and Troubadour, dry matter concentration in the cultivar Alpha increased under intermittent drought stress (Levy, 1983). This may be a demonstration of the cultivar’s ability to prevent water loss by evapotranspiration due to the low surface area of its relatively small canopy (Tourneux et al., 2003). However, in this experiment, a “white course net” was used to reduce the infection of subject plants with aphid-borne potato viruses (Levy, 1983). This method had previously been demonstrated to reduce available solar radiation by 18% (Marco, 1981), which may have disproportionately countered the purported advantages of large canopies with respect to drought tolerance (Schittenhelm et al., 2006).

Grafting experiments have shown that potato scions have a greater effect on the relative partitioning of dry matter into tubers than root stock (Jefferies, 1993a). Dry matter was preferentially partitioned into the canopy in Cara scion grafts, compared to Désirée; corresponding with greater canopy expansion, but lower tuber dry matter, under drought stress (Jefferies, 1993a). In contrast, a positive association between stem length and tuber dry matter has also been shown under conditions of total water deprivation (Deblonde and Ledent, 2001). This relationship was weak (*R*^2^ = 0.53; significant at *P* < 0.1), and only observed in one of the two trial years (Deblonde and Ledent, 2001). The experimental design also included using a “strong plastic sheet” to exclude rainfall from the droughted plots which was placed directly on the soil surface (Deblonde and Ledent, 2001). Holes were cut in the sheet for the plants at 50% emergence but, the weight of this sheet could have had a stunting effect on stems emerging later, potentially confounding the relationship between stem length and tuber dry matter. Regardless, these data could be evidence of a dominant effect of canopy architecture on drought tolerance in potato, but the nature of this relationship and the trade-offs between relative assimilate partitioning, canopy radiation interception and evapotranspiration remain unknown.

### Effects of Drought on Potato Quality

There is little previous research directly observing the effects of drought stress on physical defects in potato. This is perhaps because the primary measure of quality in processing cultivars is dry matter concentration (Pritchard and Scanlon, 1997) which has been covered above. However, structural defects have been shown to occur after even short periods of drought stress (van Loon, 1986). Intense periods of drought stress followed by heavy rainfall or irrigation during tuber bulking result in higher rates of misshapen tubers than continuous drought stress throughout the bulking phase (van Loon, 1986). Secondary growth can occur after tuber water potential drops to −500 kPa for as little as three days as intra-tuber irregularities in the conversion of assimilates into storage products causes variable growth rates across the tuber (Moorby et al., 1975). Why tubers grow uniformly before drought stress but irregularly after it is unknown, but drought stress may induce irregular intra-tuber maturation patterns which, when resupplied with water, lead to faster rates of tuber bulking in less mature areas of the tuber.

The effects of drought stress on secondary growth in potato may be confounded by the effects of temperature, which has been demonstrated to cause secondary growth regardless of drought stress (Bodlaender et al., 1964). This study also found no secondary growth in plants grown at 16°C which were subject to repeated bouts of total water deprivation, lasting several weeks, followed by an unknown amount of water (Bodlaender et al., 1964). It’s unclear whether these results are because of drought stress on secondary growth or the effects of temperature on variables not measured in this experiment; slower evaporation rate, slower growth and altered water-use efficiency could all confound the effects of low temperature and drought stress on secondary growth.

The effects of drought stress on the prevalence of misshapen tubers in potato may be cultivar dependent. The previous research is unclear on the cultivar/s used, but a more comprehensive analysis of nine cultivars found no association between drought intensity and the prevalence of misshapen tubers, except in the cultivar Kondor (Levy, 1986b). Kondor showed significantly higher rates of misshapen tubers under moderate and severe drought conditions compared to plants with an “adequate” water supply, ∼39, ∼42, and ∼23%, respectively, (Levy, 1986b). This response was only seen in the spring, not in the summer when the rate of misshapen tubers was <5% across all water treatments but average and maximum temperatures were higher (Levy, 1986b), further complicating the relationships between drought stress, temperature and tuber quality in potato.

The suggestion that the effects of drought on tuber quality are highly cultivar dependent is supported by research in the cultivar Russet Burbank, where no significant differences were found in tuber processing quality or the rates of internal and external defects across fourteen different irrigation protocols (Martin et al., 1992). These protocols included irrigated only, rainfed only and irrigated and rainfed plots as well as early, middle and late drought conditions (Martin et al., 1992). In both middle drought protocols, there was only a slight increase in external defects, which occurred at an average rate of 11.8% compared to an average of 7.3% in control plots (Martin et al., 1992). These middle droughted plots were maintained with soil water deficits of 88 mm and 135 mm after tuber initiation for the majority of tuber bulking, compared to an average of 50 mm across control watering protocols (Martin et al., 1992). These results are also supported by evidence in Russet Burbank, which found a slight increase in misshapen tubers, but only with severe drought, where available soil moisture was reduced to 25% during early tuber bulking (Painter and Augustin, 1976). The differences were again very small with “bottlenecks and dumbbells” rising from ∼12% of tubers in less severely droughted plants to ∼15% in severely droughted plants (Painter and Augustin, 1976).

It has been difficult to discriminate the effects of drought stress, temperature, and cultivar-environment interactions on structural defects in potato. While there is evidence that specific cultivars do respond to drought stress by producing misshapen tubers (Levy, 1986b), the differences in prevalence of misshapes between drought stressed and irrigated plants are small, often insignificant and may occur only with very severe drought conditions (Painter and Augustin, 1976; Levy, 1986b; Martin et al., 1992). Evidence in this area is limited and there has been little recent work investigating the effects of drought stress on structural defects in potato. This may be because a consensus seems to have been reached that temperature is the primary cause of structural defects in potato (Sparks, 1958; Bodlaender et al., 1964; Levy, 1986b; van Loon, 1986; Struik et al., 1989) but, with temperatures and incidences of drought set to rise this relationship may need more up-to-date analysis.

## Biological Strategies to Reduce the Effects of Drought Stress in Potato

The above literature outlines the general effects of drought stress on the cultivated potato, *S. tuberosum*. However, as already noted, there are many important differences between potato cultivars, not least in terms of drought tolerance. Drought tolerance in potato is mediated by complex, often poorly understood, relationships between a range of physiological and morphological variables which are affected by both genotype and environment (Spitters and Schapendonk, 1990). These variables include cultivar maturity class (Deblonde et al., 1999; Tourneux et al., 2003; Aliche et al., 2018), genetics (Schafleitner et al., 2007; Anithakumari et al., 2011, 2012), and morphology (Steckel and Gray, 1979; van Loon, 1981; Schittenhelm et al., 2006; Iwama, 2008). Here we primarily focus on potato morphology with the intention of informing future research exploiting recent developments in multispectral, three-dimensional imaging and HTP platforms.

### Drought Escape Versus Drought Tolerance

The growing season of potatoes is primarily determined by local temperature ranges throughout the year (Haverkort, 1990). To avoid the winter frosts (Sukumaran and Weiser, 1972), the United Kingdom’s lowland growing season typically begins between late-March and early-April, and ends around the end of September (Haverkort, 1990). This long season increases the probability that a period of, at least, mild drought stress will occur. Three primary biological strategies have emerged to mitigate the effects of drought on potato yields: drought escape, tolerance and avoidance (Kooyers, 2015).

#### Drought Escape

Drought escape, the simplest of these strategies, involves the rapid progression of a plant through its life cycle, decreasing the probability that drought will occur at any stage before the plant can reproduce (Muthoni and Kabira, 2016). In potato agriculture, this may be achievable by the use of early maturing cultivars which have shorter life cycles than second-early and maincrop cultivars (Griffith et al., 1984). Using early maturing cultivars to escape late season drought in a Mediterranean climate has been suggested based on crop modeling (Haverkort and Goudriaan, 1994). Experimental evidence has shown that the early maturing cultivars, Russet Norkotah (Stark et al., 2013), Blanka and Monalisa (Levy, 1986a), can escape drought stress when it occurs late in the season. However, early season droughts are much more damaging to early cultivars than those with longer life cycles, which are better able to recover once soil water is replenished (Deblonde et al., 1999). Early cultivars also produce lower overall yields than later cultivars under favorable conditions (Levy, 1986a; Stark et al., 2013) and mild drought stress (Deblonde et al., 1999). The use of drought escape for maintaining yields during drought stress is therefore an inherently high-risk, low-reward strategy while the onset, or absence, of drought within the growing season remains unpredictable.

#### Drought Tolerance and Avoidance

Drought tolerance in crop species is the ability of a plant to maintain biomass, growth or yield when exposed to drought (Tardieu et al., 2018). This vague definition has previously been used to include drought escape, described above (Tardieu et al., 2018), and drought avoidance, which involves preventing drought stress in the plant tissue despite a droughted environment (Kooyers, 2015). The difference between drought tolerance and avoidance can be considered one of scale rather than kind.

Drought tolerance is the ability of plants to weather periods of drought stress through physiological adjustments, including increased osmoprotectant production, osmotic regulation and sugar accumulation (Kooyers, 2015). Drought avoidance is the ability of plants to withstand drought through morphological adjustments, including increased root growth, stomatal closure and increased root: shoot ratio (Kooyers, 2015). Both involve increasing water use efficiency (WUE) and can be difficult to distinguish. One review of drought avoidance strategies in herbaceous populations describes root growth in response to drought as an example of both drought tolerance and drought avoidance (Kooyers, 2015). Unlike drought escape, drought tolerance and avoidance strategies are more likely to be linked, as morphological responses must be triggered by physiological changes in signaling. For example, in potato, stomatal conductance (a drought avoidance trait) responds to ABA accumulation in the leaves (Tekalign and Hammes, 2005). Therefore, in this review drought tolerance and drought avoidance strategies will both be referred to as drought tolerance.

### Potato Plant Architecture and Drought Tolerance

#### Potato Root Architecture and Drought

As stated previously, the susceptibility of potato to drought stress has been at least partially attributed to its shallow root system (van Loon, 1981). The primary function of all plant roots is to take up water and dissolved nutrients from the soil (Zwieniecki et al., 2002). It follows that some metric describing plant roots would therefore be an important predictive factor for plant growth or tuber yield, particularly under conditions where water is limited (Manschadi et al., 2008). In potato, cultivars that are more tolerant of drought stress have previously been shown to have deeper rooting systems (Steckel and Gray, 1979; Zarzyńska et al., 2017) or higher root dry weights (Lahlou and Ledent, 2005; Iwama, 2008).

**FIGURE 1:**
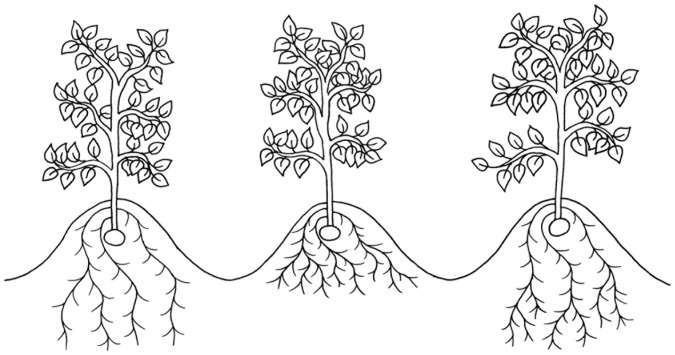
An illustration of three root morphotypes which have been suggested to improve drought tolerance in potato: deep roots (left), dense roots in shallow soil strata (middle) and dense roots in deep soil strata (right).

Previous research primarily suggests root depth is the metric most strongly associated with drought tolerance (Steckel and Gray, 1979; Lahlou and Ledent, 2005; Puértolas et al., 2014). In the seminal field study observing yields of potato cultivars with known differences in root morphology, deeper rooting cultivars, illustrated in Figure 1, were observed to maintain significantly higher yields under drought stress (Steckel and Gray, 1979). However, the authors suggest that the differences in root depth between cultivars were too small (∼100 mm) to account for the differences in drought tolerance. A later field experiment corroborated these findings with different cultivars (Lahlou and Ledent, 2005). They found a significant positive correlation between cultivar root depth at 78 DAP and a drought tolerance index, expressed as a ratio of cultivar tuber dry mass under drought to tuber dry mass with irrigation. Despite this, differences in root depth were similarly small, *R*^2^ was low (0.4956), and there was no correlation between root depth and yield under irrigated conditions.

Later, Puértolas et al. (2014) suggested that high root density at depths of >40 cm was more important to drought tolerance than root depth alone, shown in Figure 1. They suggest that small differences in rooting depth can account for the differences in yield seen in previous experiments as dense roots in deeper soil strata have greater access to ground water and thus are responsible for a disproportionate amount of water uptake. This was supported by data showing that the deepest 5% of total root length accounted for over half of the water uptake of the cultivar Cara under prolonged drought conditions (Stalham and Allen, 2004). Root growth has also been shown to be preferentially maintained over shoot growth under drought conditions, further supporting the importance of root length for drought tolerance (Jefferies, 1993a).

Root dry mass has been shown as both positively (Lahlou and Ledent, 2005) and negatively (Tourneux et al., 2003) associated with tuber yields under drought stress. Tourneux et al. (2003) experiments showed a highly significant negative correlation between root dry mass and yield, suggesting a trade-off between root production and tuber bulking which favors the former under drought conditions. In contrast, Lahlou and Ledent (2005) found a weak positive correlation between tuber yield and root dry mass under drought stress (*R*^2^ = 0.3446) and propose that the conflicting results may be due to differences in the cultivars used between the experiments.

However, the assumption that the relative drought sensitivity of potato compared to other crops is due to its comparably shallow root system has been questioned (Iwama, 2008). High intra-crop variability in root length makes it unclear which crops have the deepest and densest roots (Iwama, 2008). This may be particularly true for potato, as potato cultivars have been shown to have highly variable root systems which react differently to drought stress (Tourneux et al., 2003; Lahlou and Ledent, 2005). While this may be the case, a comprehensive comparison of root characteristics in a range of field grown crops found potato had the lowest total root length per unit area of any of the observed crops (21 km m^–2^), less than one quarter that of wheat (86 km m^–2^) (Yamaguchi et al., 1990). However, this study used only one potato cultivar, Danshakuimo (Irish Cobbler), which has been demonstrated to produce particularly shallow and short root systems, with low total dry weights (Iwama, 2008), when compared to several other cultivars (Iwama, 1998).

Due to the above associations between cultivar root length and drought tolerance, it has been suggested that root length and vigorous root growth should be prioritized as a selection criteria for breeding new, drought tolerant cultivars (Iwama, 2008; Puértolas et al., 2014). Root pulling resistance has been identified as a potential measure to select for root length in potato, and has been shown to positively associate with yield under drought conditions (Ekanayake and Midmore, 1992). This may be due to tolerant cultivars being better able to maintain, or improve, root proliferation under drought conditions, as has been shown in maize (Westgate and Boyer, 1985). The ability of cultivars to increase their root: shoot ratio under drought conditions has also been associated with drought tolerance, although its effects on yield have not yet been observed (Jefferies, 1993a).

#### Potato Canopy Architecture and Drought

The relationships between canopy characteristics of potato cultivars and drought tolerance are less well understood (Schittenhelm et al., 2006). Most research on potato canopy traits is more concerned with the effects of drought on the canopy, which have been considered above, rather than the effects of canopy traits on drought tolerance. This is understandable as drought stress affects all plants by limiting stable photosynthetic productivity at the chloroplast, leaf and canopy levels (Jones and Corlett, 1992). However, potato canopies have an important role in regulating evapotranspiration (Vos and Groenwold, 1989), dry matter partitioning (Jefferies, 1993a) and tuber yield (Schittenhelm et al., 2006) under drought conditions.

In the absence of drought or disease, the productivity of potato is linearly related to its capacity to intercept solar radiation (Allen and Scott, 1980). Thus, vigorous early canopy growth creating maximal ground coverage before tuberization has been suggested as a selection criteria to improve yield (Moll and Klemke, 1990). However, when season-long water availability cannot be guaranteed, these canopy characteristics may become suboptimal. In the absence of drought, the optimum LAI for tuber production has been placed at 4.6 (Harper, 1963), although some variation between cultivars exists (Gordon et al., 1997). However, under drought conditions, optimum total LAI becomes dependent on the trade-off between maximizing photosynthesis and minimizing evapotranspiration (Schittenhelm et al., 2006).

Compared to other crops, stomatal control of evapotranspiration rate in potato is highly conservative (Sadras and Milroy, 1996). The early closure of stomata in potato contributes to its drought sensitivity by reducing photosynthesis and assimilate production, thus reducing canopy growth and yield (Dalla Costa et al., 1997). Leaf thickness has been proposed as a drought tolerance associated trait in potato (Schittenhelm et al., 2006) as it may improve stomatal regulation of evapotranspiration (Chaves et al., 2002). This relationship has not been shown experimentally in potato, but has been shown in other agricultural species including wheat (Hameed et al., 2002), olive (Bacelar et al., 2004) and mulberry (Guha et al., 2010).

**FIGURE 2:**
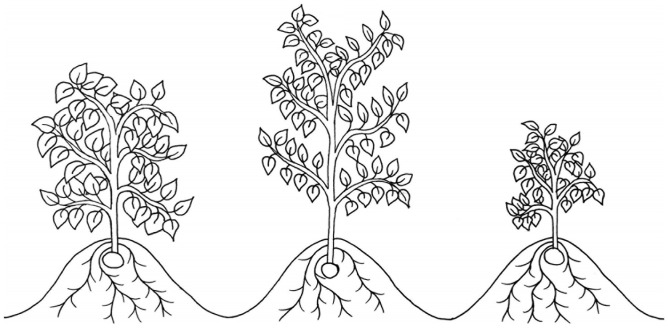
An illustration of three canopy architectures, two of which have been suggested to improve drought tolerance in potato: open “stem-type” canopies, e.g., cv. Tomba, which may improve light penetrance and interception (middle), and very small canopies, e.g., cv. Alpha, which may reduce evapotranspirative water loss (right). A dense “leaf-type” canopy, e.g., cv. Procudent, which has been suggested to be detrimental to potato yields under drought is also illustrated (left).

Small canopies may also contribute to drought tolerance in potato by reducing the surface area available for water loss by evapotranspiration (Tourneux et al., 2003), shown in Figure 2. The cultivar Alpha has been shown to produce very small canopies characterized by an average height of 10 cm, consisting of 2.5 stems with only 8.5 leaves on the main stem (Tourneux et al., 2003). This would appear to be a negative strategy for productivity considering the association between yields and solar radiation interception (Allen and Scott, 1980). However, yields in the cultivar Alpha were unaffected by drought stress, even when its water supply was incrementally decreased for 5 weeks followed by total water deprivation until plant death (Tourneux et al., 2003). This result is not merely a function of Alpha maintaining already low yields under well-watered conditions, as may be the case in Baillie, Duke of York and Ulster Scepter (Jefferies and MacKerron, 1993). The yield of Alpha under both irrigated and drought conditions was comparable to other cultivars under irrigation, including the cultivar Waycha which produces a significantly larger canopy than Alpha (Tourneux et al., 2003). This suggests reducing evapotranspiration through methods excluding stomatal closure my contribute to maintaining yields under drought stress in potato.

Leaf density has also been associated with drought tolerance in potato, with less dense stem-type canopies performing increasingly better than denser leaf-types as drought severity intensifies (Schittenhelm et al., 2006). Cultivars described as stem-type have relatively small leaf: stem ratios compared to those described as leaf-types (Schittenhelm et al., 2006), illustrated in Figure 2. But, despite having a sparser canopy, the stem-type cultivar Tomba has been shown to produce higher yields under drought stress than Procudent, a leaf-type cultivar (Schittenhelm et al., 2006). Leaf-types produce larger leaves than stem-types which, while increasing radiation interception in the short-term, can lead to self-shading (Schittenhelm et al., 2006). This results in photosynthetic inactivity in the lower leaves, which may be responsible for the yield losses of leaf-types under drought conditions (Schittenhelm et al., 2006). Stem-types have also been shown to compensate for their small leaf size by producing large open canopies (Schittenhelm et al., 2006), favored in other crops for improved light penetration (Duvick and Cassman, 1999; Murchie et al., 1999). However, it may be that the drought tolerance of Tomba is more a function of a large root mass (Schittenhelm et al., 2006) than its stem-type canopy architecture. The later hypothesis may have been supported by a more recent study, where Tomba was found to be the most drought tolerant cultivar out of seventeen, despite having the highest water consumption under well-watered and drought stressed conditions (Meise et al., 2019). Maintaining a high water consumption, even under water-restricted conditions, seems more consistent with the maintenance of a large root mass than with a stem-type canopy architecture, although, as the experiment took place in 5 l pots (Meise et al., 2019), this may not have been the case and canopy architecture cannot be ruled out as a causal factor. In another experiments, the leaf-type cultivar Konyu-2 out yielded others with similar root systems but lower leaf: stem ratios (Deguchi et al., 2010). This was attributed to the unique ability of Konyu-2 to preferentially partition dry matter into leaves over stems, allowing it to achieve an optimal LAI even under drought conditions (Deguchi et al., 2010). Due to the significant effects of root characteristics on drought tolerance outlined above and the difficulty in controlling these variables, the optimal canopy architecture for drought tolerance in potato remains unclear.

## Conclusion

Despite its status as the most profitable crop produced in many countries, particularly in the United Kingdom and central Europe (Petrenko and Searle, 2016), many morphophysiological processes of potato remain unstudied. As climate change increases the risk of summer droughts in many parts of the world (Daccache et al., 2012), an understanding of modern cultivar-environment interactions will be needed on which to base further research. Until recently, high profit margins have masked inefficiencies in potato production (Taylor et al., 2018), and perhaps reduced the emphasis on fundamental and actionable research investigating potato production. This review has highlighted the many gaps that remain in the understanding of key morphophysiological processes in potato. It is well documented that potato is a highly susceptible to drought stress (van Loon, 1981; Schafleitner, 2009; Aliche et al., 2018) but the relative effects of premature stomatal closure and reduced RuBP production on photosynthetic rate in potato remain unknown, as do the mechanisms by which stomatal conductance is regulated during severe drought stress. This has made it difficult to evaluate the optimum canopy structures for high yields under drought conditions. An understanding of the role of stomatal conductance as a drought stress response is essential for evaluating the potential trade-off in canopy size between small canopies, which reduce water loss by transpiration (Tourneux et al., 2003) and large canopies, which maximize radiation interception (Allen and Scott, 1980). The optimum potato canopy for assimilate partitioning may also factor into this trade off, as scion grafts dominate partitioning under drought stress (Jefferies, 1993a). These knowledge gaps may not have been investigated based on the assumptions that potatoes will continue to be profitable regardless and that drought tolerance in potato is adequately understood. Much of the research cited in this review states clearly and with conviction that shallow root systems are the primary cause of the drought susceptibility of potato (van Loon, 1981; Ekanayake and Midmore, 1992; Zarzyńska et al., 2017; Aliche et al., 2018; Chang et al., 2018). While root depth is associated with drought tolerance, the authors of studies investigating the relationship between root depth and drought tolerance suggest that the correlations are too weak and the effect sizes too small to account for the variation in drought tolerance seen between cultivars (Steckel and Gray, 1979; Lahlou and Ledent, 2005). In contrast, the effects of drought stress on canopy growth in potato are much more variable than on its effects on root growth (Boguszewska-Mańkowska et al., 2020). Thus, screening for drought tolerant cultivars by observing the canopy architecture of potato under drought stress will likely be faster, more convenient, and higher resolution than the less sensitive, delicate, and labor-intensive process of measuring root growth (Zarzyńska et al., 2017). However, unlike in other crops (Susič et al., 2018; Asaari et al., 2019; Zovko et al., 2019), very little work has been conducted with potato that utilizes modern phenotyping methods, such as multispectral, hyperspectral or three-dimensional imaging. These technologies present an opportunity to better understand the effects of drought stress on potato and will be a useful to accelerate the screening of drought tolerant cultivars.

Similarly, tuber quality has been almost entirely attributed to high temperatures in the field (Bodlaender et al., 1964), despite evidence in specific cultivars to the contrary (Painter and Augustin, 1976). High inter-cultivar variability in drought tolerance has been repeatedly demonstrated in potato (Steckel and Gray, 1979; Levy, 1983; Sprenger et al., 2015; Aliche et al., 2018), making it difficult to generalize the observed effects of drought on one or a few cultivars to the commercial population. Studies investigating tens of cultivars are extremely valuable (Jefferies, 1993b; Jefferies and MacKerron, 1993; Luitel et al., 2015; Aliche et al., 2018), but remain scarce due to the obvious logistical problems associated with large scale field trials. This highlights the need for a greater understanding of specific phenotypic traits, with respect to drought tolerance, which may be generalizable between cultivars with similar morphologies. Enhancing drought-protective morphological traits may then become the focus of breeding programs within *S. tuberosum* subsp. *tuberosum*, and novel traits observed in *S. tuberosum* subsp. *andigenum* may be introduced into commercial cultivars. Many of the new cultivars already produced by breeding programmes in recent decades will also need to be investigated with respect to drought tolerance. Much of the research cited here is now relatively old and would benefit from a rejuvenation of interest in drought tolerance in potato, which is becoming increasingly important as the climate changes in favor of drier growing seasons in many places.

## Author Contributions

DH produced all text, tables, and figures. DN provided expertise and reviewed the text. JH provided supervision and reviewed the text. LB provided supervision, reviewed the text, and obtained project funding. All authors contributed to the article and approved the submitted version.

## Conflict of Interest

DN was employed by the company Branston Ltd. The remaining authors declare that the research was conducted in the absence of any commercial or financial relationships that could be construed as a potential conflict of interest.
