# Corrigendum: Morphophysiology of Potato (*Solanum tuberosum*) in Response to Drought Stress: Paving the Way Forward

**DOI:** 10.3389/fpls.2021.675690

**Published:** 2021-03-30

**Authors:** Dominic Hill, David Nelson, John Hammond, Luke Bell

**Affiliations:** ^1^School of Agriculture, Policy and Development, University of Reading, Reading, United Kingdom; ^2^Branston Ltd., Lincoln, United Kingdom

**Keywords:** drought, stress tolerance, climate change, crop morphophysiology, food security, potato, *Solanum tuberosum* L., high-throughput phenotyping

In the original article, there was a mistake in the legends for Figures 1 and 2 as published. **The figure legends are correct but have been attributed to the wrong figures. The illustration comprising Figure 2 should be first in the paper with the legend from Figure 1, and vice versa**. The correct legends appear below.

**Figure 1:**
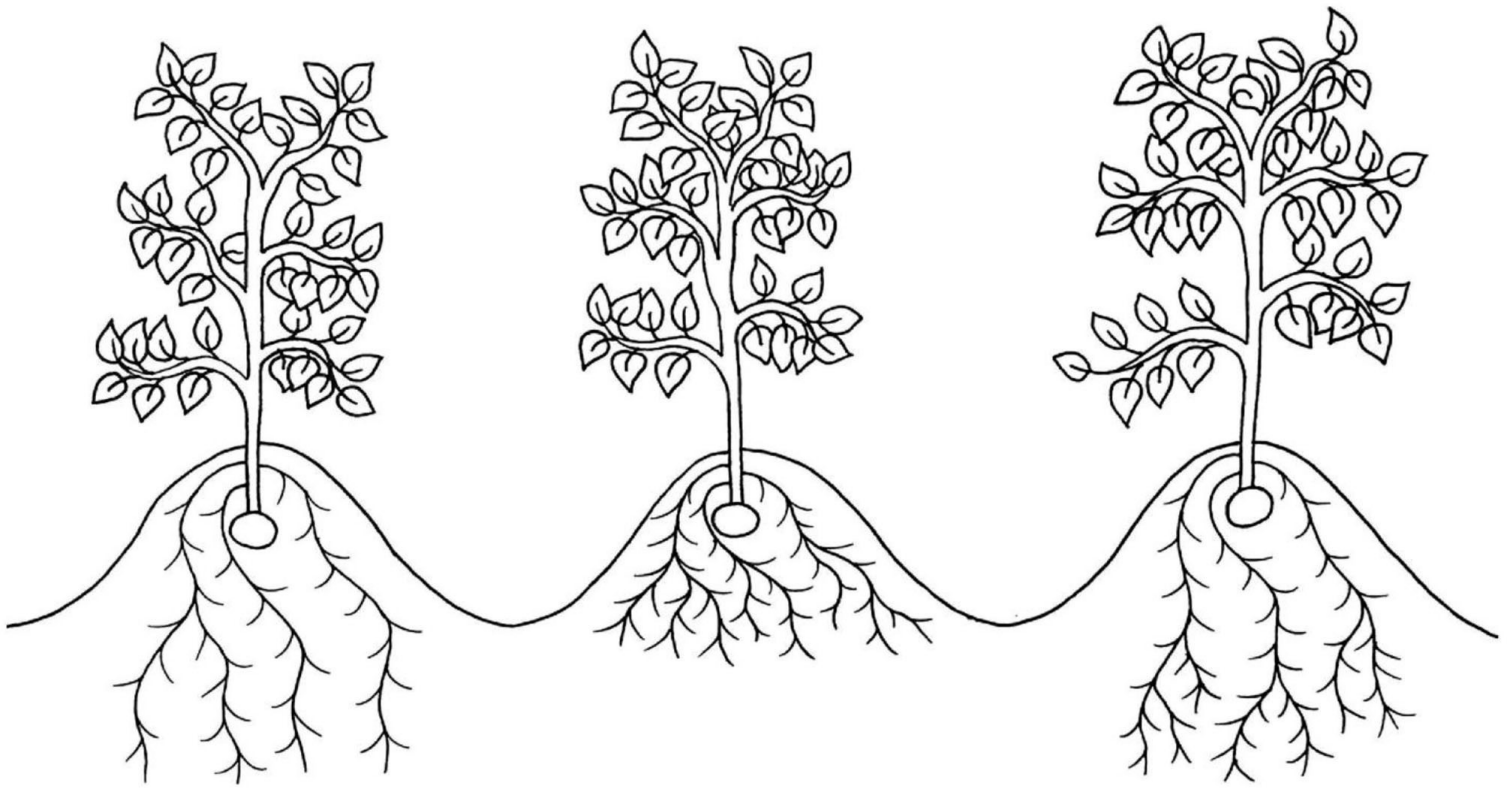
An illustration of three root morphotypes which have been suggested to improve drought tolerance in potato: deep roots (left), dense roots in shallow soil strata (middle) and dense roots in deep soil strata (right).

**Figure 2:**
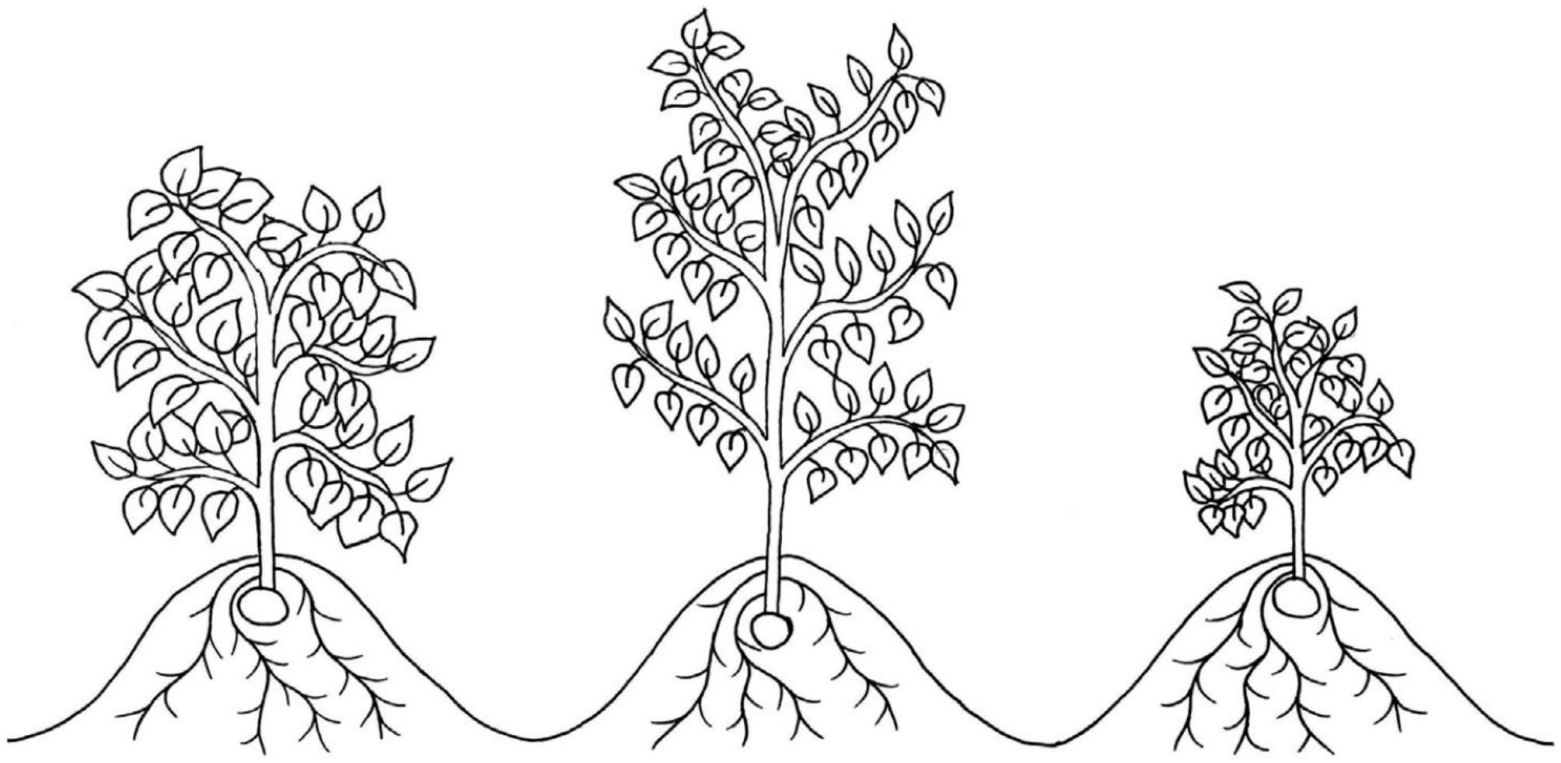
An illustration of three canopy architectures, two of which have been suggested to improve drought tolerance in potato: open “stem-type” canopies, e.g., cv. Tomba, which may improve light penetrance and interception (middle), and very small canopies, e.g., cv. Alpha, which may reduce evapotranspirative water loss (right). A dense “leaf-type” canopy, e.g., cv. Procudent, which has been suggested to be detrimental to potato yields under drought is also illustrated (left).

In the original article, there were two errors. Two **dates were printed with commas. 1807 and 1845 were printed as 1,807 and 1,845 respectively**.

A correction has been made to ***Introduction***, ***Potato Cultivation***, ***Paragraph 1***:

The cultivated potato, Solanum tuberosum, originated in the New World, where its wild relatives can still be found from the southern United States (38°N) to Argentina and Chile (41°S) (Spooner et al., 2004). Potato cultivation began in South America around 8,000 years ago (Lutaladio and Castaldi, 2009), resulting in the many thousands of landraces still grown by Andean smallholders (Bradshaw and Ramsay, 2009). Potatoes were first introduced to Europe in the 16th century by Spanish conquistadors during the Columbian exchange (Lutaladio and Castaldi, 2009). By the end of that century, potatoes had been introduced into the United Kingdom and Ireland, where they had a transformative effect on society, helping to feed the industrial revolution (Bradshaw and Ramsay, 2009). Records of potato breeding in Europe begin around a 100 years later in 1807 (Bradshaw and Ramsay, 2009), but overreliance on a few cultivars and clonal propagation resulted in the infamous destruction of the Irish potato crop by late blight in 1845 (Lutaladio and Castaldi, 2009). A concerted effort to produce resistant, high-yielding cultivars followed, some of which are still grown today (Lutaladio and Castaldi, 2009).

The authors apologize for this error and state that this does not change the scientific conclusions of the article in any way. The original article has been updated.
